# As Editor-in-Chief, I would like to acknowledge and thank, also on behalf of the Associate Editors, all the reviewers who have dedicated their time and expertise to the AE&M in 2019. The names of those who have reviewed more articles are highlighted in bold.

**DOI:** 10.20945/2359-3997000000235

**Published:** 2020-03-04

Abreu, Gabriela

Abushouk, Abdelrahman Ibrahim

Alves, Michelle

Amr, Sami S.

Andrade, Fernanda

Araújo Jr, Mário Lúcio

**Avasiloaiei, Andreea**

Baiocchi de Carvalho, Kenia

Bala, Cornelia

Bancos, Irina

Barbosa, Flavia Amanda

Batista, Thiago

Bauer, Andrea

Benseñor, Isabela

Beretta, M

Bertoluci, Marcello

Bidlingmaier, Martin

Bikle, Daniel

Bilge, Ugur

Biscolla, Rosa Paula

Bittencourt, Jackson

Blume, Carina

Bock, Patricia

Bohrer, Betânia

Bouillon, Roger

Brito, Vinícius

Bueno-Junior, Carlos Roberto

Bulcão, Caroline

**Camacho, Cleber**

**Cenci, Maria Claudia**

**Ceolin, Lucieli**

Cernea, Claudio Roberto

Cerutti, Janete

Chachamovitz, Dhiãnah

Chaves, Narriane

Chen, Ying

Chiwanga, Faraja S.

Comim, Fabio

Conceição, Flavia

Costa-Barbosa, Flávia

Cristovão, Luis

Cureau, Felipe

Cury, Adriano

Czepielewski, Mauro

Dalen, Jan Willem van

Danilovic, Débora

de Lima, Marco Antônio

de Paula, Francisco

de Paula, Leila

De Paula, Tatiana

Delfim, Ricardo

Delvecchio, Maurizio

Diaz, Rene

**Dora, Jose Miguel**

Dragovic, Gordana

Dreyer, Patrícia

Dualib, Patricia

Dumic, Miroslav

Elias, Lucila

**Elias, Paula**

Façanha, Cristina

Fayh, Ana Paula

Ferraz, Carolina

**Fighera, Tayane**

Fleseriu, Maria

Furtado, Cristiana

Fuziwara, Cesar

**Gabbay, Monica**

Gallagher, Christopher

Giudici, Kelly

Goemann, Iuri

Grob, Francisca

Guerra, Andrea

Gus, Miguel

Haddad, Luciana

Helal, Lucas

Hettiaratchi, U. P. K.

Holick, Michael

Janovsky, Carolina

Jeddi, Sajad

John, Angela

Jorgensen, Jens Otto

Kelishadi, Roya

Kim, Mimi

**Kochi, Cristiane**

Kopacek, Cristiane

Kopchick, John

Koury, Josely Correa

Kraemer de Aguiar, Luiz Guilherme

Lamounier, Rodrigo

Laurent, Michael

Lavie, Carl J.

Lehnen, Alexandre

Leitao, Marcus

Leite, Silmara

Lemos, Natália

Lemos, Sofia

Lerario, Antonio

Li, Hong

Lin, Yung-Song

Lindenau, Juliana

Lindsey, Susan

Liu, Dongfang

Liyanage, Dr. Ruvini

**Londero, Thiza**

Longo-Mbenza, Benjamin

Lou, Qingqing

Luis Zagury, Roberto

Macellaro, Danielle

Machado, Marcio

Machry, Rafael

Maciel, Gustavo

Madeira, Miguel

Malerbi, Domingos

Marcadenti, Aline

Marino, Emerson

Martins, Clarissa

**Matos, Leandro**

Mazeto, Gláucia

McKenna, Malachi

Michigami, Toshimi

Milinkovic, Neda

Mistry, Kinnari N.

Montenegro Jr, Renan

Montenegro, Ana

Montenegro, Fabio

Moreira, Carolina

Motta, Alicia

Moura, Fabio

Nader, Ana Carolina

Nascimento, Marilza

Negrato, Carlos Antonio

**Nogueira, Célia Regina**

Nordenstrom, Anna

Nunes, Vania

Ognibene, Dayane T.

**Ohe, Monique**

Oliveira, Monica

O'Neill, Ann Marie

Osorio de Castro, Luiz Felipe

Padovani, Rosália

Paes, Antonio Marcus de Andrade

**Parr, Leonardo**

Pechmann, Luciana

Penna, Gustavo

Pereira, Rosa Maria

Pinto, Jorge

Pinto, Lana

Premaor, Melissa

Preziosa, Chiara

Punales, Marcia

Rados, Dimitris

Ramos, Almino

ramos, ramon

Rassi, Nelson

**Reichelt, Angela**

Reis, Fernando

Reis, Janice

Ribeiro-Filho, Fernando

Rizzolli, Jacqueline

Rodrigues, Ticiana

Rogol, Alan D

Romitti , Mirian

Rosa, Pedro

Rosario, Pedro

Rossi, Maria Elisabeth

Sader, Soraya

Salvatori, Roberto

Santos, Alicia

**Santos, Betânia**

Satake, Eiichiro

**Scheffel, Rafael**

Schtscherbyna, Annie

Severo, Mateus

Shohaimi, Shamarina

Silva, Dalisbor

Silva, Mariana

Soares Jr, Joseé Maria

Speiser, Phyllis

Spinola-Castro, Angela

Steembugo, Thais

Stoian, Anca Pantea

Sumita, Nairo

Tavares, Ana Beatriz

Teló, Gabriela

**Texeira, Patricia**

Thomazelli, Fulvio

Tziomalos, Konstantinos

Uruska, A.

Uyar, Seyit

Valerio, Cynthia

van der Lely, Aart Jan

van Vugt, Jeroen L. A.

Varlamov, Elena

Velasco, Ines

**Velloso, Licio**

Viana, Luciana

Vianna, André

**Villagelin, Danilo**

von Frankenberg, Anize

Ward, Laura

Weinert, Letícia

Wender-Ozegowska, Ewa

Wildemberg, Luiz Eduardo

Wilkinson, Thomas J.

Wiltgen, Denusa

WrÃblewska-Seniuk, Katarzyna

Yilmaz, Nafiye

Yuen, Kevin

Zagury, Roberto

Zajdenverg, Lenita

Zantut Wittmann, Denise

